# Social and ecological determinants of antimicrobial resistance in Africa: a systematic review of epidemiological evidence

**DOI:** 10.1017/ash.2024.375

**Published:** 2024-09-09

**Authors:** Catherine Bennett, Will Russel, Rebecca Upton, Frank Frey, Bineyam Taye

**Affiliations:** 1 Department of Neuroscience, Colgate University, Hamilton, NY, USA; 2 Global Public Environmental Health, Colgate University, Hamilton, NY, USA; 3 Department of Biology, Colgate University, Hamilton, NY, USA

## Abstract

**Background:**

Antimicrobial resistance (AMR) is one of the greatest global health problems for humans, animals, and the environment. Although the association between various factors and AMR is being increasingly researched, the need to understand the contribution of social and ecological determinants, especially in developing nations, remains. This review fills these knowledge gaps by synthesizing existing evidence on the social and ecological determinants of AMR in Africa.

**Results:**

Twenty-four studies were selected based on predefined criteria from PubMed. 58.33% (n = 14) and 29.17% (n = 7) of the studies reported on ecological and social determinants of AMR, respectively, and 3 (12.5%) studies documented both social and environmental determinants of AMR. Sociodemographic factors include increased household size, poor knowledge, attitudes toward AMR, low educational levels, and rural residences. Indicators of poor water sanitation and hygiene, framing practices, and consumption of farm products were among the common ecological determinants of AMR and AM misuse in Africa.

**Conclusion:**

Our review demonstrates the importance of social and ecological determinants of AMR among African populations. The findings may be valuable to researchers, policymakers, clinicians, and those working in lower-income countries to implement AMR prevention programs utilizing a holistic approach.

## Introduction

Antimicrobial resistance (AMR) occurs when microbes grow in the presence of a drug whose purpose is to eliminate these microbes.^1,2^ The rise of AMR has been catalyzed by the overuse of antimicrobial treatments in humans and animals^3,4^ and unsuccessful efforts to develop new alternative antimicrobials.^5,6^ AMR exacerbates negative health outcomes in patients, resulting in higher mortality rates and extended hospital stays.^7^ As a result, labor supply and productivity diminish.^8,9^ Currently, AMR is one of the leading causes of infection-related deaths worldwide, exceeding annual deaths due to tuberculosis, malaria, and HIV/AIDs combined.^7,10^ It is estimated that by the year 2050, AMR will be responsible for 10 million deaths per year worldwide, exerting a total global cost of US$100 trillion.^11,12^ Although AMR affects all nations,^10,13,14^ developing nations are disproportionately affected.^7,15^ Of all Global Burden of Disease Study regions, sub-Saharan Africa (SSA) and South Asia had the highest mortalities (23.5 deaths per 100,000 and 21.5 deaths per 100,000, respectively) attributable to AMR.^16^ Generally, weaker infrastructure in these regions coincides with a high burden of infectious diseases, inadequate prevention measures, and other social determinants that contribute to high AMR rates.^17,18^ In SSA, there is little regulation of the prescription and sale of antimicrobials,^19^ poor adherence to treatment guidelines by professionals, and inadequate access to culture and sensitivity tests to identify drug-resistant bacteria.^20^ Although AMR surveillance systems are an integral part of public health, chronic underfunding and weak infrastructure are the most common problems in African countries.^21^ Less than half of these countries are enrolled in the WHO Global Antimicrobial Resistance and Use Surveillance System,^22^ and even fewer have implemented AMR guidelines and reported surveillance data.^20^

Public health intervention efforts to address AMR focus on a “One Health” approach, which emphasizes the role of social and ecological determinants in creating systemic barriers to AMR.^23–25^ This approach includes efforts to reduce the burden of disease; improve food consumption, water, sanitation, and hygiene access; and provide knowledge about antimicrobials.^26^ Studies in high-income countries have demonstrated how the examination of social and ecological factors reveals macro-level trends that can be monitored and addressed through intervention.^27,28^ In contrast, studies in SSA focus on the magnitude of AMR but provide limited information to address social and ecological factors that contribute to it. Previous systematic reviews on AMR in Africa summarize the magnitude of AMR and its ecological distribution,^29,30^ and a few reviews have synthesized social determinants trends of AMR in Africa.^21,31^ To the best of our knowledge, no systematic review in Africa has examined both the social and ecological determinants of AMR. Researchers and decision-making bodies must be aware of the social and ecological influences on AMR and account for how they are intertwined to identify meaningful targets and implement effective interventions.

## Methods

### Literature search and selection

Predefined inclusion and exclusion criteria were used to identify relevant epidemiological studies using the PubMed database (Supplementary Table 1). To be considered, studies had to meet the following conditions: (i) publication in English; (ii) published within the past 10 years (after 2013); (iii) a cohort, case-control, cross-sectional study, or randomized controlled trial; (iv) identified social or ecological determinant exposures through interviews, hospital records, Area Deprivation Index, survey data, health-related social needs screening tools, livestock samples, water analysis, air quality assessments, soil examinations, and other relevant social or ecological factors; and (v) AMR determination using methods such as antimicrobial susceptibility testing, whole genome sequencing or metagenomics, bioinformatics, microarrays, commercial antibiogram techniques, immunochromatography, polymerase chain reaction (PCR) testing, MALDI-TOF mass spectrometry, AMR surveillance, or other applicable methods. Studies that appeared as letters, editorials, reviews, correspondences, and case reports were excluded from the analysis. Study eligibility was independently assessed by two researchers (CB, WR) through a meticulous 3-step process, beginning with a title examination, followed by an abstract review, and concluding with a full-text evaluation.

### Study quality assessment

The quality of studies was assessed using an adapted version of the Newcastle–Ottawa assessment scale (NOS) for observational studies.^32^ The evaluation was based on 3 criteria: the selection of study groups, the comparability of groups, and the confirmation of exposure and outcomes. Cross-sectional and cohort studies were considered to be high quality if they had a score of 7 or greater out of 10.

### Narrative analysis

Table 1 synthesizes the studies that were categorized as either social or ecological as determined by the exposure measurement. Thematic analysis resulted in the subcategories of social determinants of AMR (n = 7): demographic information, education history and awareness of AMR, study settings (urban vs rural), and the economic low-resource setting status of participants. The ecological determinants (n = 14) were categorized into themes such as water sanitation and hygiene (WASH), pollution, farming practices, food safety, water analysis, and wastewater treatment. Studies that discussed both social and ecological determinants (n = 3) had a combination of at least 1 social and 1 ecological determinant.

**Table 1.** Summary characteristics of studies included

First author, year	Country	Study design	Outcome measures	Exposure measures	Type of sample population	Category of determinant	Region of Africa	Sample number	NOS
Afema, 2016	Uganda	Cross-sectional	Disc diffusion assay, latent class analysis, genotyping, pulsed-field gel electrophoresis (PFGE)	Water samples, livestock samples,	Environmental water samples, livestock samples, and human wastewater samples	Ecological	East Africa	N=602	7
Caudell, 2017	Tanzania	Cross-sectional	AM use and human exposure to AM	Surveys	Humans	Social	East Africa	N=415	7
Markkanen, 2023	Benin, Burkina Faso, and Finland	Cross-sectional	Metagenomic sequencing	Water samples	Hospital wastewater	Ecological	West Africa	N=79	8
Bougnom, 2019	Cameroon and Burkina Faso	Cross-sectional	Metagenomic sequencing, bioinformatics	Water samples	Raw sewage samples	Social and ecological	West Africa	N=6	8
Muloi, 2022	Kenya	Cross-sectional	Metagenomic sequencing, bioinformatics	Fecal samples, survey	Human, livestock, and wildlife fecal sample and epidemiological surveys	Social and ecological	East Africa	N=1316 fecal sample; N=99 surveys	7
Dela, 2023	Ghana	Cross-sectional	MALDI-TOF mass spectrometry, antimicrobial susceptibility testing (AST), PCR testing	Food samples, human samples, water samples	RTE (ready-to-eat) food, washing water, storage water, cooking water, vendor palm swabs	Ecological	West Africa	N=179 RTE food, 10 vendor palm swabs, 72 water samples	5
Hamiwe, 2018	South Africa	Cross-sectional	PFGE, PCR,	Water samples	Wastewater treatment plant, surface water, hospital sewage	Ecological	Southern Africa	N=48	7
Rasool, 2021	Zanzibar	Cross-sectional	rRNA gene analysis, plastic polymer analysis, Minimal inhibitory concentration (MIC)	Plastic litter	Plastic litter	Ecological	East Africa	N=20	6
Quarcoo, 2022	Ghana	Cross-sectional	MALDI-TOF mass spectrometry, AST, PCR testing	Food samples	Lettuce	Ecological	West Africa	N=25	7
Messele, 2017	Ethiopia	Cross-sectional	PCR, disc diffusion test	Livestock	Raw beef, mutton, chicken and chevon meat	Ecological	East Africa	N=292	7
Machado, 2014	Guinea-Bissau	Cross-sectional	Antimicrobial disc susceptibility test and PCR	Water samples	Well water	Ecological	West Africa	N=4	6
Caudell, 2018	Tanzania	Cross-sectional	AST	Survey data, stool, and food samples	Human stool samples, milk samples, milk containers	Ecological	East Africa	N=391 surveys, N=226 human stool samples, N=181 milk samples, N=191 milk container swabs	7
Muleme, 2023	Uganda	Cross-sectional	EBSL chromogenic agar containing EBSL supplement	Survey, environmental, animal, and human samples	Survey, animal fecal per rectal samples, human urine and stool samples, and doorknobs, soil, animal feeding equipment, and water	Ecological	Southeastern Africa	N=196 participants from 104 households, N=393 animals samples	8
Sutherland, 2019	Rwanda	Cross-sectional	AST by Kirby-Bauer disk diffusion method	Human samples, hospital data	Wound, blood, urine, sputum samples, and hospital data	Social	East Africa	N=647	8
Ita, 2022	Kenya	Cross-sectional	Chromogenic agar plates, AST using the VITEK®2 instrument	Human samples, hospital data	Nasal and fecal samples and hospital data	Social	East Africa	N=1999 community residents, N=1023 hospital participants	9
Mongi, 2022	Tanzania	Cross-sectional	Antimicrobial use questionnaire	Survey data	Human	Social and ecological	East Africa	N=420 consumers for consumer awareness, N=60 farmers (30 eggs and 30 for Chinese)	7
Sneddon, 2022	Ghana	Cross-sectional	Survey data	Behavioral factors	Human	Social	West Africa	N=12	7
Alhaji, 2018	Nigeria	Cross-sectional	Survey data	Survey data	Human	Social	West Africa	N=384	8
Omulo, 2021	Kenya	Cross-sectional	Mac agar plates and AST	Survey, human, and water samples	Stool sample, hand swab, and drinking water	Ecological	East Africa	N=1516 interviews, N=6674 samples	9
Shawa, 2022	Zambia	Cross-sectional	MICs, genomic DNA, phylogenetic analysis, PlasmidFinder	livestock	Cloacal swabs from hens	Ecological	South-Central Africa	N=200	8
Datok, 2020	Nigeria	Cross-sectional	AST by Kirby-Bauer disk diffusion method, Multiple Antibiotic Resistant (MAR) index determination	Food samples	Suya (barbeque beef)	Ecological	West Africa	N=300	7
Beshiru, 2020	Nigeria	Cross-sectional	PCR, MAR index	Food samples	RTE shrimp	Ecological	West Africa	N=1440	7
Fuhrmeister, 2023	Global	Ecological Study	Antimicrobial resistance gene identified by Comprehensive Antibiotic Resistance Database; metagenomes	Survey, fecal metagenomes	Survey data, human fecal samples	Social	Global	N=36 studies, N=1589 metagenomes	8
Maina, 2020	Kenya	Cross-sectional	Bacterial speciation and antibiotic susceptibility tests	Hospital data	Humans	Social	East Africa	N=3590	9

## Results

### Characteristics of included studies

**Figure 1.**
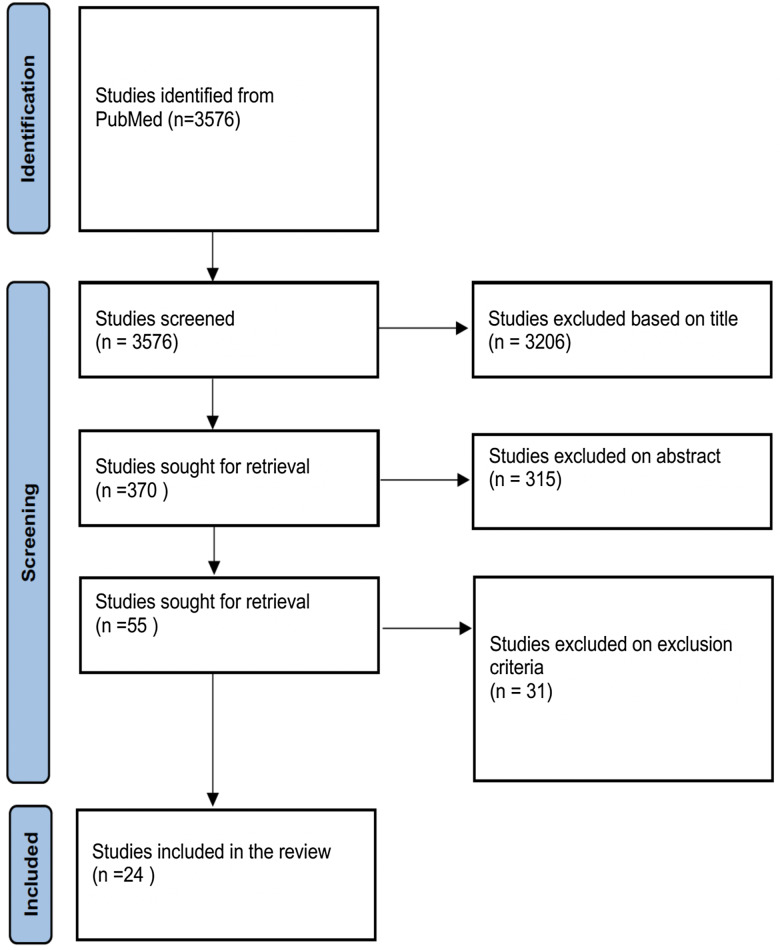
Flow diagram of systematic review selection criteria.

A total of 24 studies were used in the systematic review, all of which were retrieved from PubMed (Figure 1). With the exception of one study, all studies used a cross-sectional study design (Table 1). Of the studies examined, 45.83% (n = 11) were conducted in East Africa, 37.5% (n = 9) in West Africa, and 16.67% (n = 4) in other regions in Africa (Supplementary Figure 1). No studies were located in North Africa. Overall, 58.33% (n = 14) of the studies focused on ecological determinants of AMR, 29.17% (n = 7) on social determinants of AMR, and 3 (12.5%) discussed both social and ecological determinants of AMR.

#### Quality appraisal results

Of the 24 studies assessed, 4 were deemed of moderate quality, and 23 were of high quality (Table 1). No studies received a poor-quality assessment.

#### Social determinants

##### Household and population size

There is some evidence that overcrowded living environments at the household and population level increase the prevalence of AMR and the abundance of antimicrobial resistance genes (ARGs). A study from Kenya showed that human carriage of ARGs from *Escherichia coli* in households that keep livestock was positively associated with increased household size.^33^ A metagenomic study on raw sewage in Cameroon found that the abundance of clinically relevant ARGs was higher in a city of 3.5 million inhabitants than in a city of 1.0 million.^34^ Specifically, 71 ARGs were common between the 2 cities, but an additional 61 ARGs were unique to the larger city, with only 4 ARGs unique to the smaller city.^34^

##### Pastoralist communities

Pastoralist communities may be at increased risk of AMR, given their close proximity to livestock and their use of prophylactic antimicrobials. A study in Nigeria showed that most (60%) pastoralists purchased antimicrobials from veterinary drug shops (as opposed to “animal drug hawkers”), and two-thirds of these pastoralists practiced arbitrary applications, rather than following the instructions given.^35^ In addition, most pastoralists did not know of AMR (67%), and few (30%) thought that antimicrobial misuse in livestock could result in the emergence of resistant pathogens.^35^ Low education, lack of funds for veterinary care, seasonal movement, and nonenforcement of antimicrobial distribution laws were all strong predictors of antimicrobial misuse.^35^

A study from Tanzania highlights the complexity of understanding patterns of antimicrobial use in livestock against the backdrop of variations in cultural and livelihood practices.^36^ Ethnic groups varied in how often they sought veterinary consultations (Chaaga = 96%, Arusha = 45%, Maasai = 36%), which was inversely related to the use of self-administered antimicrobials (Chaaga = 1%, Arusha = 21%, Maasai = 74%). Additionally, all pastoralists self-administering antimicrobials did not observe the recommended withdrawal period from consumption of milk and meat. Maasai, who purchased over-the-counter medicines from veterinary drug shops, were less likely to consult with a veterinarian for livestock care. Arusha, owning transportation or a cell phone or with higher levels of education, were more likely to self-administer antimicrobials. Maasai, who owned a radio or who had electricity, were more likely to observe posttreatment withdrawal from the consumption of milk and meat.^36^ In a follow-up study,^37^ AMR from human stool samples was highest for Maasai and Arusha households and lowest for Chagga households, with >40% of isolates resistant to ampicillin, tetracycline, trimethoprim, sulfamethoxazole, and streptomycin in Maasai and Arusha households, compared to 20%–30% resistance in Chagga households.^37^ However, antimicrobial use in humans and livestock was not associated with resistance to any antimicrobial.^37^

##### Rural versus urban

Four studies explicitly assessed the impact of rural versus urban living on antibiotic use, AMR and ARG prevalence, and knowledge of AMR. The Tanzanian study noted above^36^ found a negative association between distance to urban centers and the likelihood of consulting a veterinarian for livestock care and purchase of antimicrobials in Arusha populations but not Chagga or Maasai populations.^36^ A Kenyan study of communities and hospitals in an urban (Nairobi) and rural (Siaya) setting showed that extended-spectrum cephalosporin-resistant *Enterobacterales* were more prevalent in the urban setting (52%) compared to rural setting (45%) and in urban hospitals (70%) compared to rural (63%) hospitals.^38^ A large global burden of antibiotic resistance study including 1589 metagenomes from 26 countries showed that increased access to improved water and sanitation was associated with a lower abundance of ARGs; this association was stronger in urban compared to rural settings.^39^ A Tanzanian study on antibiotic residues in foods reported that 42% of egg and Chinese cabbage consumers were unaware of the likelihood of antibiotic residues in their food,^40^ even though 73% knew that animal waste containing antibiotics was used for cultivating fruits and vegetables and 78% knew that antibiotic residues could be harmful to humans. Educational status and urban living were linked to awareness of antibiotic residues in foods.^40^

##### Clinical-based settings

A study of patients in urban and rural hospitals in Kenya found a high prevalence of extended-spectrum cephalosporin-resistant *Enterobacterales* in nasal and fecal specimens and a very low prevalence of carbapenem-resistant *Enterobacterales* and methicillin-resistant *Staphylococcus aureus*, with a higher prevalence of resistant *Enterobacterales* in urban compared to rural hospitals.^38^ A study of a teaching hospital in Kigali, Rwanda, revealed that gram-negative bacteria are highly resistant to antibiotics that are usually prescribed. Additionally, patients who had undergone surgery, taken antimicrobials in the past 30 days, or were transferred from another hospital had a greater chance of developing AMR.^41^

In Ghana, a study exploring antimicrobial prescribing behavior and the knowledge of teams treating dental patients found that antimicrobials were viewed as a fundamental aspect of dental care and were being prescribed both therapeutically and prophylactically, with amoxicillin, metronidazole, and amoxicillin/clavulanic acid being the most commonly prescribed.^42^ Some respondents shared concern about the cleanliness of facilities and the sterility of instruments used as a rationale for the extensive use of antimicrobials to prevent postoperative infections. They also wanted prescribers to follow the prescription advice of senior colleagues rather than advice from the national standard treatment guidelines.^42^ Most respondents had a good general knowledge of AMR, but this knowledge did not affect their decision-making around antimicrobial prescribing, which was influenced more by the perception of the patient’s ability to pay.^42^

#### Ecological determinants

##### Water, sanitation, and hygiene (WASH)

The aforementioned metagenomic global burden of antibiotic resistance study^39^ found that the abundance of ARGs was highest in Africa, followed by Southeast Asia and South and Central America. Increased access to improved water and sanitation was negatively associated with the abundance of ARGs for tetracycline and trimethoprim but not with the abundance of ARGs conferring resistance to beta-lactams, fluoroquinolones, aminoglycosides, or streptogramins. A study in Nairobi, Kenya, showed that children eating soil and the presence of communal handwashing stations within a block of households were associated with an increased load of antimicrobial-resistant *E. coli*.^43^ However, rainfall was the best predictor of a decreased load of antimicrobial-resistant *E. coli*, suggesting that transmission through unsanitary living conditions overwhelms the incremental effects of prior antibiotic use.^43^ A study of water quality in Guinea-Bissau (West Africa) documented that samples from the wet season had 67% more fecal coliforms than samples from the dry season and had a higher proportion of samples resistant to 3 or more antibiotics (wet = 22%; dry = 9%) but a lower percentage of isolates resistant to at least 1 antibiotic (wet = 59%; dry = 72%).^44^

Several studies in different parts of Africa examined wastewater samples. In South Africa, raw sewage water had higher levels of ARGs than treated wastewater discharge.^45^ In Uganda, most (61%) *Salmonella typhimurium* and *Salmonella enteridis* isolates collected from wastewater treatment plants and slaughterhouses were susceptible to all antimicrobials tested. However, there were high levels of resistance to a variety of commonly used antibiotics, and shared *Salmonella* genotypes were found in human, livestock, and environmental sources, indicating that zoonotic and environmental transmission to humans is likely.^46^ Another study comparing the wastewater from hospitals in Benin and Burkina Faso revealed a higher relative abundance of ARGs in wastewater from hospitals in these countries compared to 6 hospitals in Finland.^47^ In Zanzibar, plastic litter from 4 rural sites that had a history of cholera outbreaks, and poor sanitation facilities contained multiple multidrug-resistant enteric pathogens, including *Klebsiella pneumonia*, *Enterobacter cloacae*, *Citrobacter freundii*, and *Vibrio cholerae*.^48^ In a Kenyan study, the availability of WASH resources like toilet handwashing stations, proper waste separation, clean toilet facilities, appropriate distance between hospital beds, and drinking water storage might explain the differences in patterns of AMR across hospitals.^49^

##### Farming

There is ample evidence of AMR associated with livestock. The Wakiso District in Uganda has the highest livestock production in the county, and when environmental, human, and livestock samples were screened for extended-spectrum-beta-lactamase-producing *E.coli*, 80% of households had at least 1 positive sample. Seventy percent had animals carrying the drug-resistant bacteria, 62% had humans carrying the drug-resistant bacteria, and 10% had environmental samples carrying the drug-resistant bacteria.^50^ The use of a protected water source for drinking, using containers with lids, and having a clean household were negatively associated with the presence of drug-resistant bacteria.^50^ A survey in Tanzania revealed that poultry farmers consistently use oxytetracycline via drinking water for disease prevention, treatment, or growth promotion, and a majority of cabbage farmers use manure from livestock treated with antibiotics to enrich their soil.^40^ Similarly, a study conducted in a Zambian poultry farm revealed that 10% of the samples contained cefotaxime-resistant *E. coli* and that all of these samples were also resistant to at least 2 other antimicrobial classes.^51^ Further analysis showed that 20% of the poultry isolates were closely related to 25% of human inpatient isolates, sharing 2 plasmids that contained 14 distinct AMR genes, pointing to transmission between poultry and humans.^51^ Another study conducted on lettuce farms in Ghana found *E. coli* in all lettuce samples, regardless of the water source used for irrigation, with most (82%) of the samples resistant to more than 1 antimicrobial.^52^ About one-fifth of raw meat samples (beef, sheep, goat, and chicken) from slaughterhouses in Ethiopia contained *E. coli* that were resistant to commonly used antibiotics, with nearly one-half showing multiple drug resistance.^53^

##### Food safety

A survey of ready-to-eat foods in Accra, Ghana, showed that *E. cloacae* was present in 17% of tested food samples and showed resistance to commonly used antibiotics in the region.^54^ Similarly, a study of ready-to-eat shrimp in Nigeria showed that 93% of ready-to-eat shrimp contained *Vibrio* species, with the most common being *Vibrio parahaemolyticus* (38%), *Vibrio vulnificus* (12%), and *Vibrio fluvialis* (10%).^55^ All isolates were 100% sensitive to colistin and gentamycin but broadly resistant to other antibiotics commonly used in the region.^55^ Likewise, a study from a different part of Nigeria showed that 13% of barbecued beef samples from local markets contained *E. coli*.^56^ All the *E. coli* strains were resistant to ampicillin, and 75% of the cultures that tested positive for *E. coli* showed resistance to multiple different antimicrobials.^56^

## Discussion

This systematic review of studies assessing social or ecological determinants of AMR in Africa points to some common themes. There is some evidence that overcrowded living environments at the household and population level could increase the prevalence of antimicrobial-resistant bacteria and the abundance of antimicrobial-resistant genes circulating in the human population. Additionally, indicators of poor water sanitation and hygiene-related variables, framing practices, and consumption of farm products were among the common ecological factors linked with AMR in Africa. It seems clear that our ability to understand patterns of AMR, and ultimately the factors driving its emergence, is going to depend on a nuanced and integrative investigation of not only factors like antimicrobial use, population density, water quality, and sanitation but also critically factors like variation in cultural practices, how livestock are raised, farming practices, and the management of healthcare facilities.

### Strengths and limitations

One of the strengths of this review was the comprehensive screening process. Eligible studies were chosen based on clearly defined inclusion criteria, and 2 reviewers (CB and BT) worked together to determine the final set of studies. This review also adds to the existing literature on the determinants of AMR by using a combination of social and ecological lenses rather than individual factors alone.

There are limitations associated with our review, however. First, PubMed was the only search engine used in our study, raising the possibility of selection bias. Another key limitation was the lack of research on social and ecological determinants of AMR in some parts of Africa, such as North Africa. Furthermore, the cross-sectional nature of the included studies in this review limits our ability to draw conclusions about the causal relationship between the exposure (social and ecological determinants) and the outcome (AMR). It is difficult to determine whether the reported AMR followed the social and ecological factors. Having this information would be crucial to establishing the temporal relationship between social and ecological factors and AMR. Although most of the included studies explored the impact of potential confounding variables on the observed association between social and ecological factors and AMR, the possibility of reverse causation is difficult to eliminate without baseline data fully. We recommend future longitudinal studies to rule out the possibility of reverse causation.

Given these limitations, our review provides an impetus to assess further complex sociodemographic and ecological factors contributing to African AMR. Furthermore, the review is particularly relevant to attempts to inform public health professionals about how social-demographic and environmental factors are interconnected and facilitate AMR in Africa.

### Implications

The principal current strategy to combat AMR among clinicians and the pharmaceutical industry is to prescribe alternative antimicrobials and develop new drugs [63]. However, this strategy is difficult to sustain. It requires ongoing monitoring and detection of AMR and testing, which makes it challenging to provide most African healthcare facilities with the resources necessary to tackle AMR effectively. Additionally, the spread of AMR bacteria is usually nonlinear and involves many social-demographic and ecological determinants. Therefore, public health experts must simultaneously design and implement holistic AMR prevention measures addressing AMR’s social and ecological determinants.

## Supporting information

Bennett et al. supplementary material Bennett et al. supplementary material

## Supplementary material

For supplementary material accompanying this paper visit http://doi.org/10.1017/ash.2024.375. click here to view supplementary material
