# Supplementary material for: Social and ecological determinants of antimicrobial resistance in Africa: a systematic review of epidemiological evidence
Source: Antimicrob Steward Healthc Epidemiol. 2024 Sep 9;4(1):e119. doi: 10.1017/ash.2024.375 (PMC11384158; doi:10.1017/ash.2024.375)
Supplement: Bennett et al. supplementary material [file S2732494X24003759sup001.docx]

**Supplementary Table 1. Search Strategy for Identifying Manuscripts**

The following search strategy, using appropriate Medical Subject Headings prefixes and suffixes were used to identify potential papers

| **#** | **Searching Terms** |
| --- | --- |
| 1 | antimicrobial resistance |
| 2 | antibiotic treatment |
| 3 | extensive drug resistance |
| 4 | pan-drug resistance |
| 5 | antibiotic resistance |
| 6 | Drug Resistance, microbial/analysis |
| 7 | Drug resistance, microbial/ drug effects |
| 8 | Drug resistance, microbial/ epidemiology |
| 9 | Drug resistance, microbial/ etiology |
| 10 | Drug resistance, microbial/ prevention and control |
| 11 | Drug resistance, microbial/ statistics and numerical data |
| 12 | Drug resistance, microbial/ therapy |
| 13 | Drug resistance, microbial/ trends |
| 14 | eco-social health |
| 15 | ecological determinants of health |
| 16 | air quality |
| 17 | ecotoxicity |
| 18 | Environment |
| 19 | Hazard exposure |
| 20 | Ecological public health |
| 21 | One Health |
| 22 | WASH |
| 23 | Hygiene |
| 24 | sanitation |
| 25 | environmental hazards |
| 26 | climate |
| 27 | soil |
| 28 | water quality |
| 29 | sewage |
| 30 | rural health |
| 31 | urban |
| 32 | social determinants of health |
| 33 | healthcare access |
| 34 | hospital access |
| 35 | socioeconomic |
| 36 | income |
| 37 | education |
| 38 | language |
| 39 | community life |
| 40 | health education |
| 41 | epidemiology |
| 42 | Foodborne pathogens |
| 43 | water contamination |
| 44 | pharmaceutical waste |
| 45 | human and animal waste |
| 46 | 1 or 2 or 3 or 4 or 5 or 6 or 7 or 9 or 10 or 11 or 12 or 13 |
| 47 | 14 or 15 or 16 or 17 or 18 or 19 or 20 or 21 or 22 or 23 or 24 or 25 or 26 or 27 or 28 or 29 or 30 or 31or 32 or 33 or 34 or 35 or 36 or 37 or 38 or 39 or 40 or 41 or 42 or 43 or 44 or 45 |
|  | **46 and 47** |

*Key to Medical Subject Headings suffixes and prefixes:*

*exp = exploded search; mp= key word;*

Search Key words

(“antimicrobial resistance” or “antibiotic treatment” or “extensive drug resistance” or “pan-drug resistance” or “antibiotic resistance” or “Drug Resistance, microbial/analysis” or “Drug resistance, microbial/ drug effects” or “Drug resistance, microbial/ epidemiology” or “Drug resistance, microbial/ etiology” or “Drug resistance, microbial/ prevention and control” or “Drug resistance, microbial/ statistics and numerical data” or “Drug resistance, microbial/ therapy” or “Drug resistance, microbial/ trends”) AND (“eco-social health” or “ecological determinants of health” or “air quality” or “ecotoxicity” or “environment” or “hazard exposure” or “Ecological public health” or “One Health” or “WASH” or “Hygiene” or “sanitation” or “environmental hazards” or “climate” or “soil” or “water quality” or “sewage” or “rural health” or “urban” or “social determinants of health” or “healthcare access” or “hospital access” or “socioeconomic” or “income” or “education” or “language” or “community life” or “health education” or “epidemiology” or “foodborne pathogens” or “water contamination” or “pharmaceutical waste” or “human and animal waste”)
